# Microbicide research in developing countries: have we given the ethical concerns due consideration?

**DOI:** 10.1186/1472-6939-8-10

**Published:** 2007-09-19

**Authors:** Keymanthri Moodley

**Affiliations:** 1 Bioethics Unit – Tygerberg Division, Faculty of Health Sciences and Centre for Applied Ethics, University of Stellenbosch, South Africa

## Abstract

**Background:**

HIV prevention research has been fraught with ethical concerns since its inception. These concerns were highlighted during HIV vaccine research and have been elaborated in microbicide research. A host of unique ethical concerns pervade the microbicide research process from trial design to post-trial microbicide availability. Given the urgency of research and development in the face of the devastating HIV pandemic, these ethical concerns represent an enormous challenge for investigators, sponsors and Research Ethics Committees (RECs) both locally and internationally.

**Discussion:**

Ethical concerns relating to safety in microbicide research are a major international concern. However, in the urgency to develop a medically efficacious microbicide, some of these concerns may not have been anticipated. In the risk-benefit assessment of research protocols, both medical and psycho-social risk must be considered. In this paper four main areas that have a potential for medical and/or psycho-social harm are examined. Male partner involvement is controversial in the setting of covert use of microbicides. However, given the long-term exposure of men to experimental products, this may be methodologically, ethically and legally important. Covert use of microbicides may impact negatively on relationship dynamics leading to psychosocial harm to varying extents. The unexpectedly high rates of pregnancy during clinical trials raise important methodological and ethical concerns. Enrollment of adolescents without parental consent generates ethical and legal concerns that must be carefully considered by RECs and trial sites. Finally, paradoxical outcomes in recent trials internationally have advanced the debate on the nature of informed consent and responsibility of researchers to participants who become HIV positive during or after trials.

**Summary:**

Phase 3 microbicide trials are an undisputed research and ethical priority in developing countries. However, such trials must be conducted with attention to both methodological and ethical detail. It is imperative that guidelines are formulated to ensure that high ethical standards are maintained despite the scientific urgency of microbicide development. Given the controversy raised by emergent ethical issues during the course of microbicide development, it is important that international consensus is reached amongst the various ethics and regulatory agencies in developing and developed countries alike.

## Background

In 1997, international debate was prompted by trials designed to prevent the vertical transmission of HIV from pregnant women to their babies [1]. This debate was advanced when HIV vaccine trials were in the planning phases and many ethical concerns were recognized [2,3]. International and national deliberation ensued and culminated in the development of international guidelines [4]. Microbicide research had an unfortunate debut with early trials paradoxically demonstrating an increased risk of contracting HIV – an outcome clearly antithetical to the objectives of such preventive research [5,6]. More recently, similar results have emerged resulting in the premature closure of the cellulose sulphate trials at five developing country sites internationally [7]. However, a wide range of microbicide  gels are in various phases of clinical trial testing necessitating on going reflection on the ethics of microbicide research internationally [8-12].

Unique ethical concerns pervade all aspects of the microbicide research process. Unlike HIV vaccine research, microbicide trials involve repeated exposure of both sexual partners to an experimental chemical product. If the product is proven to be efficacious, exposure will most likely continue on a long term basis. As such, exposure to partners and consent to such exposure is important. During the course of trials unintended pregnancy may occur, especially in trials where long term follow-up is being assessed. Safety of these products during pregnancy must be established. Adolescents, as a group, stand to benefit from the availability of an effective microbicide gel yet testing the products on them is fraught with ethical concerns. Due to the potential of microbicides to result in vaginal ulceration either as an adverse effect in normal dosage or due to overuse as self application is involved, the risk of developing HIV infection may increase rather than decrease. These concerns represent an enormous challenge for the research community and regulatory and ethics oversight agencies locally and internationally.

Most microbicide research is conducted by developed sponsor countries in developing host countries where the burden of disease due to HIV/AIDS is greatest. The Council for International Organisations of Medical Sciences (CIOMS) 2002 guideline mandates that dual review of research is conducted on all international collaborative research studies [13]. As such, the ethical concerns relating to safety are a major concern for regulatory agencies in South Africa (SA) and other developing host countries as well as for developed sponsor country Institutional Review Boards (IRBs) and investigators.

While the scientific aspects of microbicide research have been widely published [5-12,14], and extensive research relating to acceptability of microbicides and partner involvement has been conducted [15-24], there is a paucity of literature relating to the ethical deliberation around microbicide research. Ethical concerns published to date relate to treatment of HIV seroconverters during and after trials, use of placebo and condom only arms in trial design, standards of care and informed consent [25-27]. This paper addresses four main safety issues related to microbicide research where methodological and ethical concerns exist.

## Discussion

### Safety in microbicide trials

While ethical considerations range from conceptual issues in trial design through to post trial availability of efficacious products and post marketing surveillance, only the ethical issues relating to safety will be discussed in this paper. These issues include both medical and psychosocial harms that may result from microbicide use. Medical harms refer to physical adverse events that may result from the use of an experimental microbicide gel. Psychosocial harms refer to the impact of covert use of these products on relationship and family dynamics especially in developing country contexts where women are disempowered. Male partner consent remains controversial yet requires resolution to reduce both psychosocial harms as well as medical harms that could result from potential penile toxicity. Involvement of male partners becomes imperative if the inclusion of pregnant women in microbicide research becomes standard practice. This is a tangible possibility given the high pregnancy rates emerging in many microbicide trials. Enrollment of adolescents without parental consent in SA is extremely contentious and has the potential for medical and psychosocial harm to this group of vulnerable participants. An important endpoint of any microbicide clinical trial is HIV seroconversion. The expectation is that such seroconversion will be lower in the experimental microbicide gel group than the placebo group. When HIV seroconversion is higher in the experimental group, this is a cause for concern and raises questions about risk to participants and their partners, product safety, informed consent, return of results to research communities, as well as care of HIV seroconvertors. These ethical concerns will be elaborated below.

### RECs and risk-benefit analysis

RECs are charged with the weighty responsibility of participant protection in research. Central to this function is an adequate assessment of risk imposed on participants by a research study or investigational agent in relation to benefits accrued by participation. Of the wide range of products under experimentation at present, each has a different chemical composition. Risk in terms of toxicity varies from mild local symptoms to systemic absorption and risk to partners. The ethical concerns outlined in this paper will therefore apply differentially to the various investigational microbicides in clinical trial development.

Most data to date indicates a range of local vaginal side-effects. While this may sound innocuous, some adverse events of this nature have the potential for serious risk. This was demonstrated in the first microbicide trial of Nonoxynol 9 where vaginal ulceration that resulted from product use increased transmission of HIV requiring the studies to be terminated [5,6]. More recently, the premature closure of the cellulose sulphate trials was based on an unfavourable risk-benefit ratio resulting in a higher rate of HIV seroconversion in the treatment arm [7]. The first phase 1 study of an antiretroviral microbicide gel indicated that most women in the trial (92%) experienced at least one mild adverse event – these included vaginal pruritis, vaginal bruising related to applicator use, vaginal discharge, amongst others. Only one participant experienced a serious adverse event. Of note is the systemic absorption of the drug demonstrated in 14 out of 25 women. As a safety trial on a small group of sexually abstinent women and sexually active couples, over a 2 week period this trial provides important safety data for short term use of the product [14]. Long term studies will need to elucidate safety issues with chronic use. Of interest in this study is the inclusion of male partners of sexually active women. However, there is no data included on adverse events in males exposed to the gel neither is there a statement indicating that no adverse events were experienced by male participants. This is an important consideration for RECs and regulatory agencies who need to deliberate on the inclusion of male partners in phase 3 trials as well as the need to obtain agreement/consent from male partners in addition to consenting women on such trials. On the whole, there is a paucity of published information relating to effects of microbicides on males.

### Male partners – agreement/consent

The philosophy underlying microbicide trials relates to the empowerment of women to protect themselves from a life-threatening disease in settings where they are unable to negotiate safe sex. Given this rationale, it seems almost antithetical to consider obtaining agreement or consent from male partners. However, in clinical trials where both partners are exposed to an experimental vaginal gel, partner agreement/consent and couple consent as opposed to female participant consent only is an important consideration.

Several arguments have been advanced for excluding men from microbicide trials. In patriarchal communities where men are the primary decision-makers on most issues including matters relating to sexual intercourse and intimacy, researchers fear that male partners will prevent women from participating in clinical trials and from using microbicide products. In large phase 3 trials it may be impractical to get male partners to attend research sites due to competing commitments related to employment. The inclusion of men as part of phase 3 trials will significantly increase the costs and resources required for such trials in research studies that are poorly supported by the pharmaceutical industry. Finally, where female participants have multiple male partners, it may be logistically difficult to include all partners in the trial.

Equally compelling arguments can be advanced for the inclusion of male partners in microbicide trials. Firstly, male partners are being exposed to an investigational agent and are exposed to physical risk. Since pre-clinical testing of microbicides includes testing for "penile toxicity", clinical testing should also involve male partners with their consent to assess for acute and long term local effects. Failure to do so could have legal ramifications in the long term especially if chronic use of these investigational products is later found to be associated with penile toxicity or if these products are found to increase risk of HIV as has been documented in women [5-7]. In the United States, it is an FDA requirement that any microbicide with systemic activity (such as Tenofovir Gel) will require investigation for penile absorption in male partners [28]. The World Health Organisation (WHO) guidance on reproductive health research and partner involvement favours respect for individual participant autonomy in all situations. However, partner agreement should be made a condition of recruitment only if the research will "so immediately affect the partner as to make him or her comparable to a subject of the research". This is applicable to microbicide gels in general especially those with systemic absorption. As such, partner notification is justified only when there is physical risk to the partner such as infection or infertility according to the WHO guidelines [29]. These guidelines do not take into account local physical side-effects, psycho-social and emotional harm that may impact on partners exposed to experimental products.

While microbicide development has been targeted primarily at women, some acceptability studies have included men. Several of these studies have shown male partner interest in microbicide research [15-18]. In particular, research from South Africa has found that 66–82% of male partners would like to be included in microbicide trials [16].

While microbicide development has been fuelled by the hope that women will be empowered to protect themselves from their partners covertly with their own vaginal gel, many women in developing countries actually prefer to involve their partners [18-22]. This is often the case in settings where autonomy is not expressed in an individualistic sense and where communal conceptions of personhood may require couple or family consent as opposed to individual consent. In recognition of this socio-cultural preference in some developing countries the WHO, in its guidance on reproductive research, makes provision for partner agreement when required. This is, however, the exception rather than the rule as the WHO, in general, regards partner consent as inappropriate and as a violation of participant autonomy [29].

Where women prefer not to tell their partners, covert use may be very difficult. Most microbicides (86% of those being tested to date) increase lubrication [23]. Covert use is hence not possible in populations with a preference for dry sexual practices [24]. Furthermore, covert use could result in mistrust and deception in developing countries and male partners may develop antagonism towards these products that will parallel their antagonism towards male condoms. More importantly, covert use of microbicides by women could result in domestic violence. There is hence an emotional cost linked to covert use.

Finally, the testing of vaginal microbicides in pregnancy mitigates against the exclusion of male partners in the consenting process of clinical trials. The possibility of testing microbicides during pregnancy will require reconsenting women and their male partners or the father of the potential child.

### Testing microbicides during pregnancy

Most of the microbicides being tested in clinical trials are investigational products and their safety in pregnancy has not been established. Study participants are advised to be on contraception during the trial, pregnancy is monitored either monthly or 3 monthly and product use is discontinued as soon as pregnancy is diagnosed. Of the numerous microbicides in various stages of clinical testing, some are expected to be locally absorbed only while other agents, such as Tenofovir gel, have already shown evidence of systemic absorption [14].

During the conduct of microbicide trials in developing countries, it has become evident that pregnancy rates are much higher than anticipated. In the Cellulose Sulfate study there have been 50 pregnancies per hundred person years in Lagos and 21 pregnancies per 100 person years in Port Harcourt. Similar high pregnancy rates are quoted for the Savvy trials in Nigeria and Ghana [30-34]. These high pregnancy rates could impact on participant retention and the power of the study to demonstrate an effect as more women who become pregnant are taken off study product.

Several factors may account for the high pregnancy rates. Chemical pregnancies (in which implantation occurs followed by early miscarriage) may have been diagnosed as a result of an increased frequency of testing on clinical trials. Other factors include a lack of stringent criteria for contraceptive use and consequently use of unreliable contraceptive methods, inadequate counseling of participants during the informed consent process and a lack of contraceptive services at trial sites. Finally, perhaps high pregnancy rates in this group of participants is unavoidable given that most participants are at the peak of their reproductive cycles.

During current testing of microbicides unintended exposure of participants who fall pregnant has occurred. This has been anticipated and accepted by the FDA as long as specific animal testing is completed and is negative. These tests indicate whether a drug has the ability to interfere with reproductive health, fetal development and early development. Segment 1 studies test the effect of the drug on general fertility and reproductive performance in animals. Segment 2 studies look for evidence of teratology in animals and segment 3 studies examine effects on perinatal and postnatal development. For unintended exposure where women fall pregnant in spite of contraceptive use and counseling, negative segment 1 and 2 testing is required. To date, all trials in progress discontinue microbicide testing on those participants who become pregnant and where unintended exposure has occured.

Due to the high number of participants who are falling out of trials at present, the scientific validity of microbicide trials is under threat. In order to ensure trial results that will demonstrate product efficacy, continued testing of microbicides during pregnancy may be required. If intended exposure is to be permitted this will entail testing of the investigational product when women on trials inadvertantly fall pregnant. In such situations, the FDA will assess these trials on a case-by-case basis. A range of preclinical studies will be required to support microbicide testing in pregnancy. These include genotoxicity studies, general toxicology studies and carcinogenicity studies. Where segment studies are concerned, in addition to negative segment 1 and 2 studies, negative segment 3 studies will also be required. An additional consent process will be required, with increased safety monitoring pre and post delivery. At delivery, it is recommended that microbicides are stopped and recommenced 4–6 weeks after delivery [28].

To date segment 3 testing has not been conducted on microbicides. If segment 3 testing proves to be negative, microbicides could be tested on women who become pregnant on clinical trials and according to FDA regulations, these women will need to be reconsented [28].

This will pose a number of ethical issues for microbicide trials in developing countries:

1. Informed consent is difficult to obtain from non-pregnant participants on less complex trials in resource depleted settings [35-37]. Is it fair to reconsent vulnerable women with low levels of education for use of an experimental product during pregnancy? Given the complexity of the information to be provided and the comprehension of the risks involved, will the consent be truly informed?

2. If experimental use of substances is to continue during pregnancy it will be imperative to inform partners. How will this be achieved during the trial when pregnancy occurs if partners have been excluded from the consenting process when women were enrolled?

3. If terratogenicity is detected in pregnant women using the experimental microbicide, termination of pregnancy (TOP) should be an option. In countries where TOP is not legal, how can this option be provided? In countries where this is legal, who will provide this service – the trial site or state health services who are already overburdened?

4. What happens when adolescents on trials fall pregnant and continued testing is implemented? Will parental consent be required for unmarried minors?

### Adolescent participation – a case study from South Africa

The enrollment of adolescents in microbicide trials in SA has been a carefully considered decision given the very controversial guidelines in SA relating to the involvement of children and adolescents in research. Initial guidance for including children in research has been extrapolated from the Child Care Act No 74 of 1983 [38] that refers to consent for medical treatment of children. An adolescent over the age of 14 years may consent, unaided, to medical treatment (this includes HIV testing). However, for surgical treatment parental consent is required for adolescents and children under the age of 18 years. Whether a similar extrapolation will occur from the Children's Act No 38 of 2005[39] is uncertain as the Act remains silent on children and adolescents in research. According to the new Act, the age of consent for medical treatment and HIV testing has dropped to 12 years.

For research purposes, most studies, to date, have enrolled participants over the age of 18 years only without parental consent. Where HIV prevention research is concerned, Chapter 9 of the National Health Act No 61 of 2003 will apply [40]. Most sections on research in this chapter have been proclaimed by the State President in 2004 and were promulgated in May 2005. Section 71, however, has not been proclaimed as yet. This section categorises research as therapeutic and non-therapeutic. Microbicide research will be regarded as non-therapeutic. As such it will be necessary, once section 71 is proclaimed, to obtain consent from the adolescent, the parent/guardian and the Minister of Health. The guideline issued by the Department of Health in April 2005 however, categorises research on children using the criterion of minimal risk. This would then require the classification of microbicide research according to a risk level. The guideline does however encourage research amongst adolescents where needed. Given the conflicting legal frameworks on age of consent and research, RECs in SA handle these protocols on a case-by-case basis.

For example, the MCC and relevant RECs in SA have approved the participation of 16 year old girls in the clinical trial of Carraguard, without parental consent. The justification for this is based on the low age of sexual debut in SA which places young girls at high risk of contracting HIV. In other  words, adolescents would stand to  benefit from a preventive  intervention such as a microbicide. Thus, the argument for including girls younger than 18 years in microbicide research without parental consent rests on the principle of beneficence. However, a number of procedural issues in trial design have the potential to raise ethical concerns where adolescent participants are involved. In 2002, when this microbicide trial was initiated, the regulatory agency in SA – the Medicines Control Council (MCC) – specified that participants be paid R150 ($23) per scheduled trial visit. It was also a requirement of the MCC that this amount be specified in the patient information leaflet. Given the number of trial visits required in a microbicide trial extending over a 2 year period, it is possible for prospective adolescent participants to calculate that participation remuneration for the trial would amount to approximately R1500 ($214). This represents a significant inducement in poor vulnerable communities in SA. In addition, the trial requires that prospective participants are sexually active in the 3 months preceding enrollment. The incentive of remuneration related to participation has the potential to inadvertently encourage sexual activity to satisfy inclusion criteria into the trial. Another complication regarding the enrollment of adolescents on weekdays could imply that school attendance may be neglected and this would be problematic within the schooling system. It may be particularly problematic if the school authorities are aware that participation in these trials is without parental consent [41]. While it is important for adolescents to be enrolled in HIV prevention trials it is also important for RECs to establish criteria to protect these vulnerable participants.

### Paradoxical trial results

The recent closure of the cellulose sulphate trials in developing country sites globally[7] has reawakened fears similar to those elicited by the Nonoxynol 9 trials. Higher than expected rates of HIV seroconversion producing an unfavourable risk-benefit ratio to participants has generated sufficient concern to prematurely terminate cellulose sulphate trials globally. In parallel with the scientific concerns, a host of ethical concerns have emerged. Central to these is the content of the consent information given to these participants. Were participants informed that there was a possibility of a higher than normal risk of contracting HIV based on the Nonoxynol 9 trial results and if so, did participants appreciate this risk? Have all participants been traced and informed of their results? What impact does this have on current and future microbicide research? How will partners be informed that they have been placed at risk as a result of an experimental product? What about partners who may have seroconverted? What about cases of HIV seroconversion where covert use of the microbicide has occurred and male partners were not aware of their exposure to the gel and to an increased risk of HIV? These and other questions form the basis for a Department of Health inquiry into the early closure of the South African cellulose sulphate trial site.

### The way forward

When HIV vaccine research was being planned, international debate relating to the ethical concerns was initiated, several publications ensued and comprehensive national and international guidelines evolved. Microbicide research, on the other hand, has received considerably less attention from a regulatory and ethical oversight perspective.

It may be argued that inadequate attention has been devoted to the role of male partners in the research process. This is an issue that needs to be discussed and deliberated by RECs, especially in developing country contexts. The role of male partners in microbicide research presents an unparalleled opportunity for future empirical research at trial sites.

The informed consent process is pivotal in microbicide research. Issues related to pregnancy prevention need to be clearly outlined in consent documents. Reliable and safe contraceptive methods must be specified and made available at trial sites. Given the inevitability of pregnancy in the target population for microbicide trials, it is imperative that segment 3 testing on microbicides is conducted as soon as possible. In this way, it will be possible for intentional testing of microbicides to occur during pregnancy coupled with an intensive informed consent process involving male partners.

Adolescent enrollment is important. However, RECs must request a plan from trial sites regarding avoidance of coercion and interruption of schooling. If necessary, special arrangements must be made for adolescents to be seen after school or on Saturdays. Trial remuneration should not be included in consent forms. For long term studies, reduced remuneration should be considered and endorsed by RECs and regulatory agencies, especially in developing countries where small amounts of money may be regarded as coercive in settings of participant vulnerability.

The informed consent process must also be clear about the possibility of increased risk of HIV infection given the precedent set by Nonoxynol 9 and Cellulose Sulphate. In the event of such an outcome, clear procedures must be in place for tracing of participants, provision of treatment and notification of partners.

Finally, the crisis created by the premature closure of the cellulose sulphate trials must be handled with sensitivity. Responsible media coverage is essential to prevent sensationalisation of microbicide research and to minimize the negative impact on enrollment.

## Summary

The complexity of microbicide research in developing countries is augmented by a host of unique ethical concerns. In the haste to develop an urgently needed microbicide (which in itself is an ethical imperative), some of these concerns, especially those related to safety have either not been anticipated or fully explored. The development of guidelines pertaining to the ethics of microbicide research is important to investigators and ethics committees alike internationally. Both formative and empirical research is required to resolve these dilemmas to ensure participant protection without obstructing the conduct of urgent and important HIV preventive research. Reaching consensus on ethical oversight at an international level is crucial.

## Competing interests

The author(s) declare that they have no competing interests.

## Pre-publication history

The pre-publication history for this paper can be accessed here:


